# Supplementary material for: TaCKX2.2 Genes Coordinate Expression of Other TaCKX Family Members, Regulate Phytohormone Content and Yield-Related Traits of Wheat
Source: Int J Mol Sci. 2021 Apr 16;22(8):4142. doi: 10.3390/ijms22084142 (PMC8073499; doi:10.3390/ijms22084142)
Supplement: Supplementary file 1 [file ijms-22-04142-s001.zip › ijms-1159193-supplementary.pdf]

Supplementary Material

Table 1. a, b, c. Mean values of yield-related traits and ratio indicators (quotient of the mean relative values in silenced plants by values in non-silenced plants) in group of T_1 plants with predominately silenced *TaCKX2.2.2* (a), *TaCKX2.2.1* (b) and T_2 plants with highly silenced *TaCKX2.2.2* and non-silenced *TaCKX2.2.1* (c). * - significant at $p \leq 0.05$; ** – significant at $p \leq 0.01$; […] – standard error of the mean.

a) T_1

Expression Level of *CKX2.2.2* (Number of Plants)	Expression of *CKX2.2.1***	Expression of *CKX2.2.2***	CKX Activity	Plant Height (cm)	Spike Number	Spike Length (cm)*	Grain Number	Grain Yield	TGW	SPAD 1st Spike
0.41–0.77 (19)	0.59 [0.12]	0.68 [0.09]	1.05 [0.31]	59.26 [3.64]	4.26 [1.48]	9.71 [1.28]	136.26 [67.97]	6.67 [3.38]	49.04 [3.61]	50.29 [4.95]
0.80–1.55 (56)	0.76 [0.12]	1.05 [0.20]	0.92 [0.24]	60.48 [3.49]	4.48 [1.67]	10.38 [1.18]	159.90 [78.73]	7.82 [3.81]	49.43 [4.22]	50.71 [5.77]
Ratio indicator	0.78	0.64	1.14	0.98	0.95	0.94	0.85	0.85	0.99	0.99

b) T_1

Expression Level of *CKX2.2.1* (Number of Plants)	Expression of *CKX2.2.1***	Expression of *CKX2.2.2***	CKX Activity	Plant Height (cm)*	Spike Number	Spike Length (cm)	Grain Number	Grain Yield	TGW	SPAD 1st Spike
0.42–0.79 (52)	0.65 [0.10]	0.90 [0.23]	0.97 [0.27]	60.87 [3.48]	4.40 [1.69]	10.28 [1.18]	156.63 [82.52]	7.65 [4.00]	49.53 [4.46]	50.48 [5.55]
0.80–1.08 (23)	0.89 [0.08]	1.09 [0.23]	0.91 [0.24]	58.21 [3.05]	4.47 [1.43]	9.98 [1.39]	145.21 [57.13]	7.11 [2.86]	48.76 [2.64]	50.93 [5.65]
Ratio indicator	0.73	0.82	1.07	1.05	0.98	1.03	1.08	1.08	1.02	0.99

c) T_2

Expression Level of *CKX2.2.2* (Number of Plants)	Expression of *CKX2.2.1*	Expression of *CKX2.2.2*	Expression of *CKX2.1*	CKX Activity	Plant Height (cm)	Spike Number	Spike Length (cm)	Grain Number	Grain Yield	TGW	SPAD 1 1st Spike	SPAD 2 Next Spikes
a) 0.26–0.60 (25) highly silenced	1.13 [0.37]	0.49 [0.09]	1.06 [0.18]	0.94 [0.29]	72.40 [3.44]	5.64 [1.44]	8.74 [0.78]	121.32 [44.93]	4.90 [1.98]	40.28 [8.34]	45.93 [6.52]	43.68 [5.24]

b) 0.61–0.79(23) silenced	1.18 [0.30]	0.70 [0.05]	1.23 [0.27]	1.03 [0.31]	72.74 [4.31]	5.30 [2.46]	8.34 [1.43]	121.61 [80.05]	4.63 [3.34]	36.79 [8.89]	45.39 [5.42]	41.93 [5.78]
c) 0.80–1.25 (17) non-silenced	1.10 [0.27]	0.96 [0.12]	1.28 [0.32]	1.12 [0.34]	75.06 [4.09]	6.12 [1.91]	8.81 [1.23]	145.47 [67.72]	4.91 [2.19]	35.16 [8.35]	40.32 [5.05]	37.56 [5.47]
d) Control (18) R_2					71.67 [4.62]	6.56 [2.22]	9.31 [1.13]	168.83 [75.24]	5.98 [3.02]	34.51 [6.17]	45.83 [6.86]	39.15 [6.95]
Silenced (a + b)/non-silenced (c + d)					0.99	0.87	0.94	0.78	0.88	1.11	1.06	1.12
Highly silenced (a)/non-silenced (c)					0.96	0.92	0.99	0.83	1.00	1.15	1.14	1.16
Highly silenced (a)/control (d)					1.00	0.87	0.94	0.73	0.83	1.17	1.00	1.12

Table 2. a, b. Correlation coefficients between yield-related traits and *TaCKX* GFM expression (a) as well as phytohormone contents (b). Bold, red - significant at $p \leq 0.05$. Nonparametric tests are highlighting in grey.

a)

a	**Spike Number**	**Spike Length**	**Grain Number**	**Grain Yield (g)**	**TGW**	**Root Mass**	**CKX Activity**	***TaCKX 1***	***TaCKX 2.1***	***TaCKX 2.2.1***	***TaCKX 2.2.2***	***TaCKX 11***	***TaCKX 5***	***TaCKX9***
Plant height	0.42	0.63	0.61	**0.85**	0.67	0.01	**0.78**	0.56	−0.33	0.29	0.20	−0.31	0.51	0.61
Spike number		0.63	**0.88**	**0.77**	−0.16	0.40	0.01	0.22	0.34	−0.03	0.51	−0.15	0.23	0.36
Spike length			**0.81**	0.68	−0.11	0.08	0.25	0.14	0.47	0.11	0.67	−0.45	0.18	0.19
Grain number				**0.90**	0.01	0.52	0.22	0.22	0.32	−0.21	0.75	−0.32	0.16	0.34
Grain yield (g)					0.42	0.38	0.51	0.39	−0.09	−0.01	0.48	−0.29	0.38	0.61
TGW						−0.07	0.67	0.46	**−0.90**	0.25	−0.35	0.10	0.53	0.62
Root mass							0.06	−0.18	0.23	**−0.83**	0.64	0.02	−0.31	−0.26
CKX activity								0.25	−0.36	0.02	0.12	−0.45	0.09	0.23
TaCKX1									−0.21	0.50	−0.18	0.46	0.64	0.21
TaCKX2.1										−0.37	0.69	−0.41	−0.58	−0.56
TaCKX2.2.1											−0.66	0.27	0.75	0.46
TaCKX2.2.2												−0.59	−0.48	−0.21
TaCKX11													0.57	−0.07
TaCKX5														0.60

b)

b	tZ	tZR	tZGs	cZ	cZOG	cZ9G	DZR	DZGs	iP	BA	IAA	ABA	SPAD first spike	SPAD next spikes
Plant height	0.02	−0.60	0.32	−0.34	−0.64	−0.42	0.13	−0.11	0.32	0.27	−0.43	0.29	0.04	0.05
Spike number	0.27	−0.12	0.03	0.62	−0.53	−0.36	−0.32	−0.34	−0.53	**0.83**	−0.35	0.64	−0.13	−0.70
Spike length	−0.45	−0.09	−0.26	−0.09	**−0.85**	**−0.80**	−0.58	−0.57	−0.24	0.58	−0.49	**0.94**	−0.42	−0.58
Grain number	0.15	−0.14	0.26	0.20	−0.78	−0.54	−0.13	−0.41	−0.17	**0.79**	−0.41	0.72	−0.23	−0.74
Grain yield (g)	0.29	−0.54	0.41	0.06	−0.76	−0.41	−0.13	−0.17	0.10	0.67	−0.54	0.60	0.02	−0.38
TGW	0.38	−0.77	0.73	−0.50	−0.31	0.07	0.67	0.35	0.75	−0.27	−0.14	−0.20	0.41	0.61
Root mass	0.58	0.60	**0.87**	0.09	−0.22	0.05	0.47	−0.30	0.32	0.40	0.43	0.14	−0.33	−0.62
CKX activity	0.02	−0.26	0.52	−0.61	−0.12	0.06	0.49	0.13	0.54	−0.18	0.31	0.26	0.07	0.32
TaCKX1	0.04	−0.66	0.37	−0.26	0.31	0.11	0.13	−0.32	0.14	0.09	−0.71	−0.54	−0.18	0.36
TaCKX2.1	−0.47	0.49	−0.75	0.29	0.03	−0.26	**−0.80**	−0.47	−0.68	0.58	−0.09	0.60	−0.52	**−0.79**
TaCKX2.2.1	−0.33	−0.71	−0.71	0.11	−0.04	−0.30	−0.27	−0.01	−0.19	−0.04	−0.77	−0.14	0.14	0.52
TaCKX2.2.2	−0.12	0.03	0.40	−0.06	−0.46	−0.35	−0.36	−0.40	−0.18	0.58	−0.03	**0.83**	−0.40	**−0.86**
TaCKX11	0.37	−0.03	0.08	0.24	0.11	−0.08	0.40	−0.28	0.31	−0.09	−0.43	−0.77	−0.22	0.25
TaCKX5	0.14	−0.77	−0.02	0.09	−0.46	−0.48	0.22	−0.23	0.25	0.27	**−0.83**	−0.20	0.00	0.31
TaCKX9	0.30	**−0.83**	0.11	0.19	−0.59	−0.12	−0.04	0.42	0.05	0.27	−0.54	0.26	0.67	0.20
tZ		0.20	0.76	0.47	−0.01	0.47	0.58	0.30	0.33	0.22	0.43	−0.31	0.35	0.00
tZR			−0.20	0.30	−0.10	−0.09	0.13	−0.31	−0.30	0.07	0.60	0.00	−0.66	−0.54
tZGs				−0.36	−0.15	0.33	**0.85**	0.17	**0.93**	−0.13	0.50	−0.10	0.11	0.11
cZ					0.08	0.17	−0.65	0.05	−0.77	0.68	−0.10	0.26	0.24	−0.41
cZOG						**0.93**	0.39	0.48	−0.19	−0.78	0.30	**−0.89**	0.10	0.43
cZ9G							0.45	**0.76**	−0.01	−0.58	0.54	−0.71	0.57	0.40
DZR								0.04	**0.85**	−0.39	0.65	−0.65	0.04	0.53
DZGs									0.02	−0.58	0.54	−0.09	**0.95**	0.52
iP										−0.44	0.30	−0.10	−0.03	0.47
BA											−0.51	0.54	−0.22	**−0.76**
IAA												−0.10	0.09	−0.09
ABA													0.14	−0.71
SPAD 1st spike														0.44

Table 3. Sequence queries (%) and identities (%) between the sequence used in RNAi construct (highlighted) and each *TaCKX* gene family member located to the genomes A, B and D.

	TaCKX	*TaCKX2* [Query%/Ident%]
1.	*TaCKX1*-3A TraesCS3A02G109500	-
2.	*TaCKX1*-3B TraesCS3B02G128700	-
3.	*TaCKX1*-3D TraesCS3D02G111300	-
4.	*TaCKX2.1*-3A TraesCS3A02G311000	[99%/87,93%]
5.	*TaCKX2.1*-3B TraesCS3B02G161100	[99%/91,49%]
6.	*TaCKX2.1*-3D TraesCS3D02G143600	[99%/92,27%]
7.	*TaCKX2.2.1*-3A TraesCS3A02G311100	[99%/94,30%]
8.	*TaCKX2.2.1*-3B TraesCS3B02G161000	[99%/93,25%]
9.	*TaCKX2.2.1*-3D TraesCS3D02G143500	[99%/92,99%]
10.	*TaCKX2.2.2*-3D TraesCS3D02G143300	[99%/99,48%]
11.	*TaCKX2.2.3*-3D TraesCS3D02G143200	[99%/91,26%]
12.	*TaCKX3*-1A TraesCS1A02G159600	-
13.	*TaCKX3*-1B TraesCS1B02G176000	-
14.	*TaCKX3*-1D TraesCS1D02G157000	-
15.	*TaCKX4*-3A TraesCS3A02G481000	-
16.	*TaCKX4*-3B TraesCS3B02G525300	-
17.	*TaCKX4*-3D TraesCS3D02G475800	-
18.	*TaCKX5*-3A TraesCS3A02G321100	-
19.	*TaCKX5*-3B TraesCS3B02G344600	-
20.	*TaCKX5*-3D TraesCS3D02G310200	-
21.	*TaCKX7*-6A TraesCS6A02G185800	-
22.	*TaCKX7*-6B TraesCS6B02G214700	-
23.	*TaCKX7*-6D TraesCS6D02G172900	-
24.	*TaCKX8*-2A TraesCS2A02G378300	-

25.	*TaCKX8*-2B TraesCS2B02G395200	-
26.	*TaCKX8*-2D TraesCS2D02G374600	-
27.	*TaCKX9*-1A TraesCS1A02G234800	-
28.	*TaCKX9*-1B TraesCS1B02G248700	-
29.	*TaCKX9*-1D TraesCS1D02G237200	-
30.	*TaCKX10*-7A TraesCS7A02G363400	-
31.	*TaCKX10*-7B TraesCS7B02G264400	-
32.	*TaCKX10*-7D TraesCS7D02G359700	-
33.	*TaCKX11*-7A TraesCS7A02G536900	-
34.	*TaCKX11*-7B TraesCS7B02G455000	-
35.	*TaCKX11*-7D TraesCSU02G106300	-

Table 4. Sequences of primers designed for amplification of the genes.

Ref 2	Ta2291R	NCBI	GCTTCTGCCTGTCACATACGC	165
	Ta2291F		GCTCTCCAACAACATTGCCAAC	
TaCKX1	TaCKX1_188R		CCCAGGTACTCCTTGTACCCTAT	188
	TaCKX1_188F		GTCTACCCGCTCAACAAATCC	
TaCKX2.2.1	TaCKX2_1_R_205		TATCACATACGCCATCCATGC	205
	TaCKX2_1_F_205		TTGATCGCGGAGCTAATCCA	
TaCKX2.2.2	TaCKX2_2_R_175		ATCGTATCCTGGCCTCCTCA	175
	TaCKX2_2_F_175		TACCCCATGAACCGGAACAG	
TaCKX2.1	TaCKX2_3_R_144		TCTCCTCGTTCTGCTCCTCC	144
	TaCKX2_3_F_144		TCTACCCCATGAACCGGGAC	
TaCKX5	TaCKX5_3B_4R		CATACATGACACCAACGTACATCTT	150
	TaCKX5_3B_4F		GTCCGATTTTTGAGAAGACTGATT	
TaCKX9	TaCKX10_R_167		ACATAAAGCAATTTACCTGGACTTG	167
	TaCKX10_F_167		GAGCTAAGGGCTTGTGGGA	
TaCKX11	TaCKX3_150R		GAATTAGAGTTCACGGCTTGATG	150
	TaCKX3_150F		TTGTCAAGGGACTGTAGTAGGG	
